# Thoracic Aortic Aneurysm and Giant Cell Arteritis: Clarifying the Link

**DOI:** 10.1055/a-2765-8610

**Published:** 2025-12-23

**Authors:** Sebastien Strachan, Mohammad A. Zafar, Sudhir Perincheri, Awab Ahmad, Nafiye Busra Celik, Mah I. Kan Changez, Bulat A. Ziganshin, John A. Elefteriades

**Affiliations:** 1 Department of Cardiac Surgery, Aortic Institute at Yale-New Haven, Yale School of Medicine, New Haven, Connecticut, United States; 2 Department of Pathology, Yale School of Medicine, New Haven, Connecticut, United States

**Keywords:** giant cell arteritis, thoracic aortic aneurysm, ascending aorta

## Abstract

**Objective:**

We aim to better define the association between thoracic aortic aneurysm (TAA) and giant cell arteritis (GCA), thereby enhancing cross-diagnosis, monitoring, and therapy.

**Methods:**

Literature review: We used a two-step search approach to the available literature on the relationship between TAA and GCA. First, databases including PubMed, Web of Science, and Embase were searched. Additionally, relevant studies were identified through secondary sources including references of initially selected articles.

Retrospective cohort study: We identified patients at our institution who were diagnosed with both TAA and GCA from January 1980 through December 2024. Descriptive statistics were used to support the association between these two diseases described in the literature.

**Results:**

The literature review disclosed an increased incidence and relative risk of TAA among patients with GCA. GCA patients experienced progressive aortic enlargement, which may be due to vascular inflammation and disruption of elastin and collagen fiber biology in the vessel wall, resulting in mechanical weakness. Progressive aortic enlargement, including the aortic annulus, often results in aortic insufficiency (AI); in surgery, complete aortic replacement is recommended. Predictors of aneurysmal disease included AI and severe inflammatory response at the time of GCA diagnosis, as well as risk factors such as male sex, hypertension, hyperlipidemia, coronary disease, diabetes, and smoking.

The investigation at our institution revealed that among 2,344 patients with GCA, 72 developed TAA, an incidence of 3.1%. Among those, 61 (84.7%) had an ascending aortic aneurysm, 5 (6.9%) had a descending aortic aneurysm, and 6 (8.3%) had both. Of these, 33 (45.8%) were male, 66 (91.7%) had hypertension, 44 (61.1%) were former or current smokers, 16 (22.2%) had diabetes mellitus, 66 (91.7%) had hyperlipidemia, 31 (43.1%) had coronary disease, 33 (45.8%) had concomitant polymyalgia rheumatica, and 21 (29.2%) had AI at the time of GCA diagnosis.

**Conclusion:**

Our study highlights a 3.1% incidence of TAA in GCA patients, with hypertension, smoking, and hyperlipidemia as the most common additional risk factors. Ascending aortic aneurysms were the most frequent, occurring in 84.7% of TAA in GCA cases. These findings emphasize the importance of monitoring for TAA in the GCA population.

## Introduction

Giant cell arteritis (GCA), also known as temporal arteritis or Horton disease, is the most common idiopathic vasculitis affecting large- and medium-sized arteries, particularly the carotid artery, its major branches, and the aorta. 1 This condition primarily affects older adults and is characterized by a chronic inflammatory response within the vessel walls. Clinically, patients with GCA typically present with systemic symptoms, such as fever and fatigue, and cranial ischemic manifestations that are directly related to vascular involvement. 1 2 3 However, there is an increasing recognition of “occult” manifestations that may not be associated with typical symptoms. 2 3

The inflammatory damage in GCA leads to the disruption of elastin and collagen fibers within the aortic wall, resulting in mechanical weakening and an increased risk of aortic aneurysms (AA) and dissections (AD). 2 4 These structural changes appear to preferentially affect the thoracic aorta, specifically the ascending segment, making thoracic aortic aneurysms (TAA) more common in this population than abdominal aortic aneurysms (AAA). 1 The reason for ascending preponderance is presently unknown.

TAAs may develop either before or at the time of GCA diagnosis but more commonly arise during the later course of the disease, often 6 to 7 years after diagnosis. 2 3 5 6 Because AAs frequently remain asymptomatic, they may not be diagnosed until a life-threatening complication, such as dissection or rupture, occurs—sometime many years after the initial GCA diagnosis. 5 7 The average time between a diagnosis of GCA and thoracic aortic dissection (TAD) is 1.1 years, in contrast to the average of 10.9 years after the diagnosis of GCA when a TAA, without rupture, is diagnosed. 4 However, the relative risk and timeline of aneurysm development in GCA patients remains unclear, with different studies reporting variable estimates. 4 8

Despite increased awareness of TAA in GCA patients, the incidence, predictive factors, role of imaging, and aortic complications in this population remain inconsistently reported across studies. This has implications for screening, diagnosis, and management strategies for aortic complications, potentially impacting patient outcomes. The aim of this literature review/clinical study is to better define the association between TAA and GCA, thereby enhancing clinical awareness of their relationship and promoting earlier TAA screening and diagnosis in GCA affected patients.

## Materials and Methods

### Literature Review Search Strategy

**Fig. 1:**
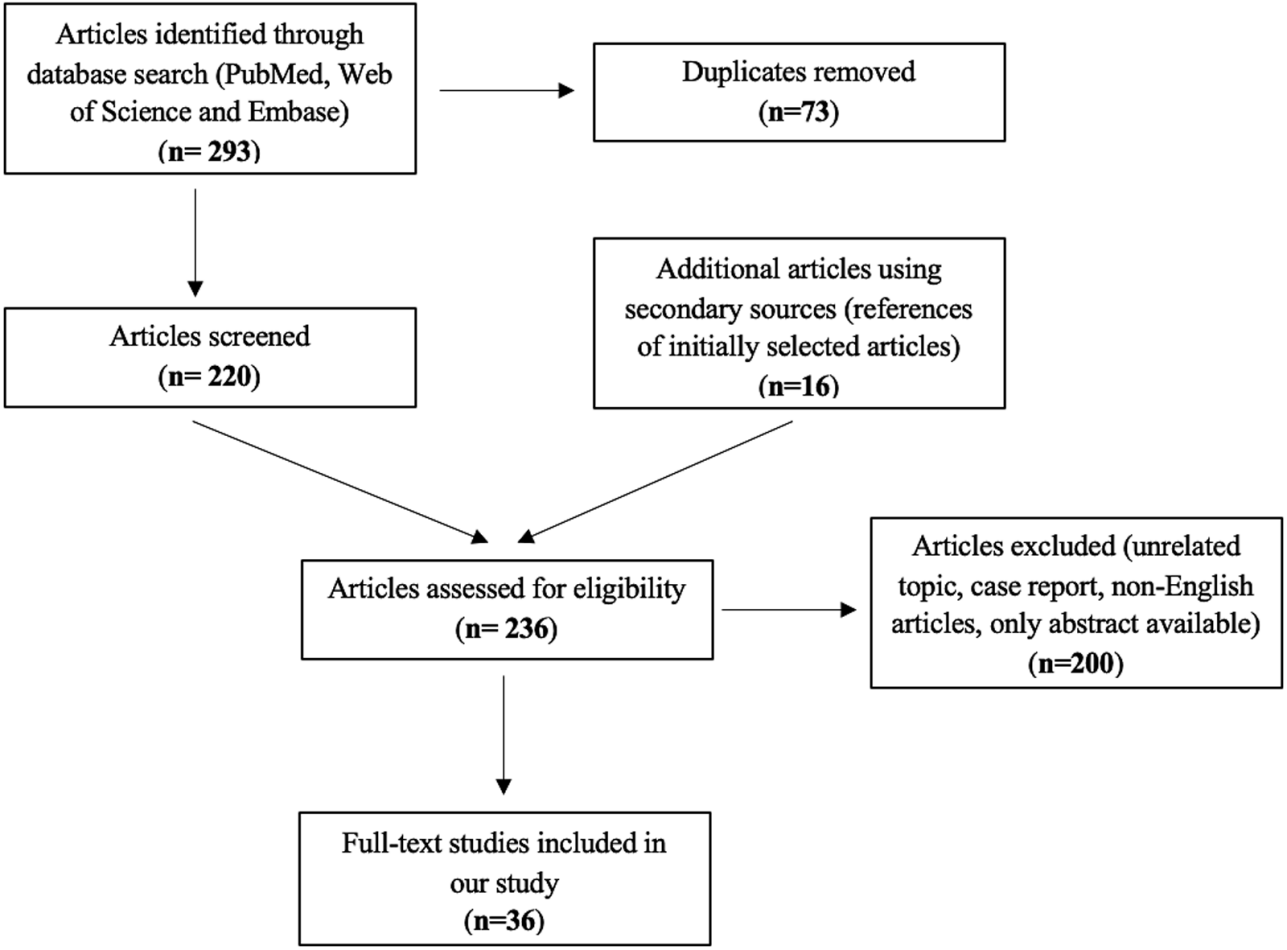
Flowchart incorporating the algorithm of search strategy.

We used a two-step search approach to the available literature on TAA and GCA. First, the search included databases such as PubMed, Web of Science, and Embase covering studies from database inception up to December 2024. We used a combination of MeSH terms including “Aortic Aneurysm, Thoracic,” “Giant Cell Arteritis,” “Temporal Arteritis,” and “Horton Disease” to capture relevant studies. Second, relevant studies were identified through secondary sources including references of initially selected articles ( Fig. 1 ). We did not follow formal PRISMA guidelines ( http://prisma-statement.org/ ) for this less formal overview.

### Retrospective Study at Our Institution

We conducted a retrospective cohort study to identify patients within the Yale New Haven Health System who were diagnosed with both TAA and GCA between January 1980 and December 2024. Patients were identified using a computer-based indexing system. Inclusion criteria required unequivocal evidence of TAA confirmed by computed tomography (CT) and/or echocardiography, as well as a diagnosis of GCA established by temporal artery biopsy, ultrasound, and/or fulfillment of the American College of Rheumatology classification criteria. 9 Descriptive statistics were utilized to examine the association between these two conditions.

## Results

### Literature Review

#### Incidence and Risk of Aortic Aneurysm and Dissection in Giant Cell Arteritis Patients

Patients with GCA have an increased risk of developing TAAs and AD. A recent meta-analysis found a 3-fold increased risk of these complications compared with controls. 4 Likewise, a UK cohort study reported a 2-fold increased relative risk of AA in GCA patients compared with age-, gender-, and location-matched controls from the general UK population. 10 The reported incidence of AA and AD in GCA patients ranges from 8.2 to 33%, depending on the length of follow-up. 4 5 11 12

Population-based studies further highlight this risk. Patients with GCA have a 17.3-fold increased risk of developing TAA and a 2.4-fold increased risk of isolated AAA compared with age- and sex-matched controls. 5 Similarly, screening studies using chest radiography and abdominal ultrasonography, followed by CT where indicated, demonstrated significant aortic dilatation in 22.2% of patients within 5 years of diagnosis. 13

Regional and national studies have also been conducted. A retrospective study in Lugo, in northwestern Spain, which examined 210 biopsy-proven GCA patients from a population of 250,000, found that 9.5% developed aortic aneurysmal disease. Among these, 16 had TAAs, whereas 6 had abdominal aneurysms. The incidence of AA and/or AD in this cohort was 18.9 per 1,000 person-years, comparable to the 18.7 per 1,000 person-years reported in Olmsted County. 3 11

Correspondingly, a Danish nationwide cohort study found that GCA patients had a significantly higher 15-year relative risk of TAA (relative risk [RR]: 11.2, 95% confidence interval [CI]: 7.41–16.9) and AD (RR: 6.86, 95% CI: 7.41–16.9) compared with the general population. 8

Importantly, the true risk of AA in GCA patients may be even higher than reported. Many asymptomatic AAs may go undiagnosed, and deaths resulting from undetected ADs could be misattributed to myocardial infarction. 7

#### Screening and Imaging for Aortic Involvement in Giant Cell Arteritis

Aortic involvement in GCA is increasingly recognized, with imaging studies showing that up to 66.7% of patients exhibit aortitis at diagnosis, as detected through positron emission tomography (PET), CT angiography, or magnetic resonance angiography. 14 15 16 17 This aortic inflammation has been associated with an increased risk of AA and AD, with PET-CT studies demonstrating the link to aortic dilation, structural damage, and aneurysm formation. 14 18

Given this elevated risk, societal guidelines recommend CT or magnetic resonance imaging of the thoracic aorta as part of the initial evaluation for all GCA patients. 19 20 However, adherence to this guideline remains inconsistent due to specialty specific “tunnel vision,” particularly among specialists such as neuro-ophthalmologists, who may not be fully aware of the potential aortic complications. 4 19

This raises an ongoing question in clinical practice: should all patients with GCA undergo screening for aortic involvement? Currently, there is no universal consensus. While the American College of Cardiology/American Heart Association/American Association for Thoracic Surgery/Society of Thoracic Surgeons and American College of Radiology advocate for baseline imaging in all newly diagnosed GCA patients, some experts suggest that screening should be determined on a case-by-case basis. 4 7 19 20 The decision to pursue additional or routine aortic imaging should be individualized based on patient-specific factors such as disease progression, treatment response, and overall risk profile. 4 20

#### The Role of Aortic Imaging in Assessing Giant Cell Arteritis Patients at Diagnosis

The utility of aortic imaging at the time of GCA diagnosis remains debated. A case–control study comparing GCA patients who underwent thoracic CT scans at diagnosis to age- and sex-matched controls hospitalized for pneumonia found no significant difference in aortic diameter between the two groups. 21 Additionally, GCA patients with aortitis did not exhibit significantly larger aortic diameters than those without aortitis at diagnosis. 21

However, findings from other studies challenge these results, reporting that aortic thickening and dilation in GCA patients are already present at the time of diagnosis. 22 23 24 Also, in a large cohort of 171 GCA patients with well-documented aortitis at diagnosis, an AA or AD was the initial presentation in 23.4% of cases, with 5.8% requiring surgery at the time of diagnosis. 25 These data support the value of performing aortic imaging at the time of GCA diagnosis.

The discrepancy among studies suggests that early aortic involvement may not be uniformly detectable and could vary depending on the stage and severity of the disease at the time of evaluation.

Despite these variations at diagnosis, progressive aortic enlargement over time is well established in GCA. In one study, the ascending, descending thoracic, and suprarenal abdominal aortic diameters increased significantly over 31 months in large-vessel vasculitis patients, while no such changes were observed in age- and sex-matched lymphoma controls. 25 These findings underscore the importance of long-term vascular monitoring in GCA patients, even when initial imaging does not reveal significant abnormalities.

#### Predictors of Large-Artery Complication (Aortic Aneurysm, Aortic Dissection) in Giant Cell Arteritis Patients

Clinical features associated with aortic dilatation or aneurysm in GCA patients include smoking, 10 26 hypertension, 3 27 and male sex. 10 13 27 28 In the previously mentioned study from the Lugo region, a pronounced inflammatory response and hypertension at the time of GCA diagnosis were found to potentially promote the development of aneurysmal disease. 3 Additionally, the presence of an aortic insufficiency (AI) murmur at diagnosis was predictive of subsequent AA and/or AD. 11 A population-based study ( *n* = 168) reported a strong association between AA or AD and the presence of hyperlipidemia (82.8%) and coronary artery disease (55.2%) at any point during follow-up ( *p* < 0.05 for both). 11 Diabetes and vascular complications—such as aortitis, atheroma, and limb ischemia—present at the time of GCA diagnosis were also identified as predictive factors for the development of vascular events, including aneurysm and/or dissection. 29

Furthermore, studies have shown that GCA patients exhibiting increased fluorodeoxyglucose (FDG) uptake in the aorta at diagnosis had significantly larger diameters of the ascending and descending thoracic aorta, along with greater thoracic aortic volume. 30 These findings suggest that increased aortic FDG uptake may identify a subset of GCA patients at higher risk for progressive thoracic aortic dilatation. 14 27 30

#### Outcomes of Patients with Aortic or Extracranial Giant Cell Arteritis

Patients with aneurysms caused by GCA face a high risk of catastrophic complications. 31 In the Olmsted County population study, 44% (4 of 9) of patients with GCA died suddenly due to AD, underscoring the severity of this condition. 5 In a series of 23 patients with GCA presenting with dissection, 46% of whom presented catastrophically; the 2-week mortality rate was 80%. 32

A retrospective review conducted at the Mayo Clinic further emphasized the life-threatening nature of thoracic aortic involvement in GCA. 31 Among 17 patients with aneurysmal disease of the descending or thoracoabdominal aorta, five died—three from complications of TAAs, including two with documented rupture and one who developed disseminated intravascular coagulation secondary to a contained thoracic rupture. Histopathologic findings commonly reveal near-complete disruption of the elastic medial layer, suggesting that dissection and rupture may occur before aneurysms reach the conventional size thresholds for surgical intervention. 31

Additionally, a review of 72 cases of aortic and extracranial GCA with histopathologic confirmation reported 18 deaths attributable to complications directly related to extracranial GCA. 33 The primary causes of death included ruptured AA (6 cases), AD (6 cases), stroke (3 cases), and myocardial infarction (3 cases).

In a separate review of 386 cases of ascending aorta and aortic valve replacement for TAA, 10 patients were identified with histopathologically confirmed GCA. 34 Annuloaortic ectasia was present in 80% of these cases, resulting in significant aortic valve regurgitation, with most patients undergoing the Bentall procedure. The authors recommended replacement of the aortic root in GCA patients, regardless of macroscopic findings due to the high prevalence of annuloaortic ectasia. Six-year survival was 90%, and 77% of patients remained free from reoperation.

The prognostic impact of aortic involvement in GCA has also been demonstrated in population-based cohort studies. One such study found that long-term survival was significantly reduced in GCA patients with AA or AD compared with the general population, highlighting the grave implications of these vascular complications of GCA. 35 Conversely, another population-based study found no overall difference in survival between GCA patients with and without large-artery involvement. However, within that cohort, patients who developed thoracic AD experienced markedly increased mortality. 36

### Institutional Study

#### Thoracic Aortic Aneurysm or Dissection in Patients with Giant Cell Arteritis

Out of 2,344 patients at our institution diagnosed with GCA between 1980 and 2024, a total of 72 patients (3.1%) were also found to have TAA, potentially related to the underlying vasculitis. This proportion may represent an underestimate, as some individuals coded with GCA may have had only a clinical suspicion rather than a confirmed diagnosis—potentially inflating the total GCA cohort and thereby lowering the apparent rate. Among those with both GCA and TAA, 29 had a positive temporal artery biopsy, 3 had a positive halo sign on ultrasound, and the remaining 40 fulfilled clinical and/or laboratory criteria sufficient to meet the American College of Rheumatology classification for GCA. 9

Of these 72 patients, 61 (84.7%) had an ascending AA, 3 (4.2%) had a descending AA, and 8 (11.1%) had aneurysms involving both segments. The most common pattern of ascending AA involved the mid-ascending segment only (55.9%). TAD occurred in 5 cases (6.8%) ( Table 1 ).

**Table 1:** Thoracic aortic aneurysm and dissection in patients with giant cell arteritis

	Patients ( *N* )	%
Thoracic aortic aneurysm	72	3.1 a
Ascending aorta	61	84.7 b
Root	2	5.9 c
Mid	19	55.9 c
Arch	2	5.9 c
Root + mid	6	17.6 c
Mid + arch	5	14.3 c
Descending aorta	3	4.2 b
Both ascending/descending	8	11.1 b
Thoracic aortic dissection	5	6.9 b

a Percentage calculated out of patients with giant cell arteritis ( *N* = 2,344).

b Percentage calculated out of patients with thoracic aortic aneurysm ( *N* = 72).

c Percentage calculated among patients with ascending aortic aneurysm, excluding those with missing ascending aortic location data ( *N* = 34).

Among the patients with both conditions, 34 were diagnosed with TAA after the diagnosis of GCA. In this subgroup, the interval between diagnoses ranged from 0.5 to 192 months (mean ± standard deviation: 57.7 ± 43.7 months; median: 50.0 months). In contrast, 20 patients were diagnosed with TAA prior to the diagnosis of GCA.

#### Risk Factors for Aortic Aneurysm in Giant Cell Arteritis Patients

Several clinical features have been identified as predictors of large-artery complications in GCA, such as AA and AD. These include male sex, smoking, hypertension, hyperlipidemia, coronary artery disease, diabetes mellitus, and the presence of AI at the time of diagnosis. 3 10 11 13 26 27 28 29 In line with these prior reports, our cohort of 73 patients with GCA and TAA showed a high prevalence of such risk factors: 33 (45.8%) were male, 66 (91.7%) had hypertension, 44 (61.1%) were former or current smokers, 16 (22.2%) had Type 2 diabetes mellitus, 66 (91.7%) had hyperlipidemia, 31 (43.1%) had coronary artery disease, and 21 patients (29.2%) had documented AI at the time of GCA diagnosis ( Table 2 ). Additionally, 33 patients (45.8%) had concomitant polymyalgia rheumatica and 7 (9.7%) had aortitis. Notably, 97.2% of patients had at least two of these risk factors simultaneously, and 88.9% had three or more, underscoring the clustering of comorbidities in this population and their potential contribution to complication risk.

**Table 2:** Predictors of aneurysmal disease in patients with giant cell arteritis and thoracic aortic aneurysm

	Patients ( *N* )	%
Male	33	45.8 a
Hypertension	66	91.7 a
Former/current smokers	44	61.1 a
Type 2 diabetes mellitus	16	22.2 a
Hyperlipidemia	66	91.7 a
Coronary artery disease	31	43.1 a
Aortic insufficiency at GCA diagnosis	21	29.2 a

Abbreviation: GCA, giant cell arteritis.

a Percentage calculated out of patients with thoracic aortic aneurysm ( *N* = 72).

#### Thoracic Aortic Outcomes and Mortality

**Fig. 2:**
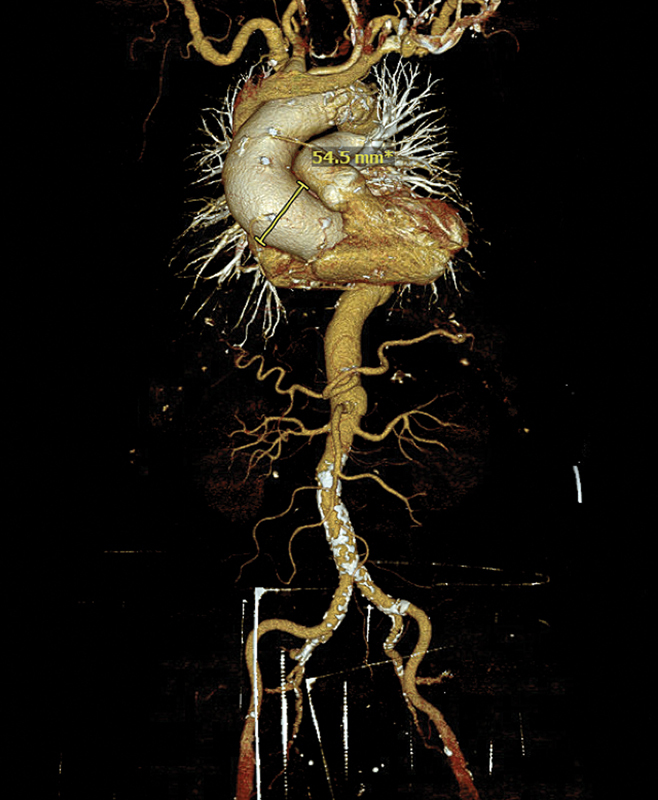
Computed tomographic angiogram of a patient with temporal arteritis and thoracic aortic aneurysm. Three-dimensional reconstruction demonstrates a large ascending aortic aneurysm measuring 54.5 mm in a patient who underwent surgical repair.

A total of 14 patients underwent TAA repair or replacement prior to rupture or dissection ( Table 3 ). Of these, 10 patients underwent ascending aortic repair or replacement, including one Bentall procedure. Two patients had descending thoracic aortic replacement; one underwent thoracic endovascular aortic repair (TEVAR), and one patient had combined ascending and descending thoracic replacement. The mean diameter of the ascending aorta at the time of surgical intervention was 5.17 ± 0.86 cm ( Fig. 2 ). Among the two patients who underwent descending aortic replacement, one was in a prerupture state due to a deep penetrating ulcer, with an aortic diameter of 3.5 cm. The patient who underwent TEVAR had an aortic diameter of 8.8 cm at the time of intervention. Among the 14 patients who underwent thoracic aortic surgery, 8 also had aortic valve repair or replacement. There were no perioperative deaths.

**Table 3:** Outcome or intervention of patients with giant cell arteritis and thoracic aortic aneurysm

Outcome/intervention patients ( *N* )	
TAA	72
Nondissecting aneurysm	68
Dissecting aneurysm	4
Death due to dissection of aorta	1
TAA repair/replacement	14
TAD repair	5

Abbreviations: TAA, thoracic aortic aneurysm; TAD, thoracic aortic dissection.

**Fig. 3:**
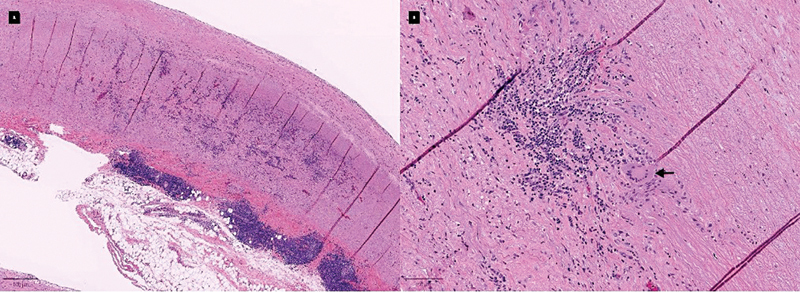
Histologic features of giant cell arteritis in resected ascending aorta. ( **A** ) Low-power view showing transmural inflammation of the aortic wall. ( **B** ) High-power view highlighting a multinucleated giant cell (arrow), characteristic of granulomatous aortitis.

Histologic analysis of resected ascending aortic specimens in select cases demonstrated transmural chronic inflammation with focal multinucleated giant cells, consistent with GCA. These findings further support the inflammatory nature of the aneurysmal degeneration observed in this population ( Fig. 3 ).

Among the five patients who experienced TAD, three were classified as Type A and two as Type B. Three patients underwent surgical or endovascular repair, one was managed conservatively, and one experienced rupture resulting in fatal hemorrhage.

For the Type A dissections, the aortic diameters at the time of dissection were 4.7 cm and 5.5 cm in two patients, whereas the third case occurred before the development of an aneurysm. For the two Type B dissections, the aortic diameters were 5.5 and 5.6 cm, respectively.

## Conclusion

By literature review and analysis of our own GCA population, we are able to demonstrate a close association between GCA and TAA. This association needs to be disseminated bilaterally: the medical community needs to be alert for TAA and to image the aorta routinely. The aortic surgical community needs to bear in mind that a population of TAA seen in practice arise not from the familiar blend, genetically triggered medial destruction, but rather from an intense inflammatory granulomatous arteritis process. Surgeons should also keep in mind that the aortic valve leaflets and the aortic annulus in GCA patients are likely affected; valve and root replacement procedure should be considered. Also, surgical patients with arteritis on pathology exams should be referred for medical and/or neurological consultation.

Both physicians and surgeons need to be aware of the powerful link between GCA and TAA. The link is strong.
